# Framework for integrating animal welfare into life cycle sustainability assessment

**DOI:** 10.1007/s11367-017-1420-x

**Published:** 2017-11-20

**Authors:** Laura Scherer, Brian Tomasik, Oscar Rueda, Stephan Pfister

**Affiliations:** 1 Institute of Environmental Sciences (CML), Leiden University, Einsteinweg 2, 2333 CC Leiden, Netherlands; 2 Foundational Research Institute, Berlin, Germany; 3 Effective Altruism Foundation, Basel, Switzerland; 4 Institute of Environmental Engineering, ETH Zurich, Zurich, Switzerland

**Keywords:** Animal products, Animal welfare loss, Diet, Food, Indicator framework, LCA

## Abstract

**Purpose:**

This study seeks to provide a framework for integrating animal welfare as a fourth pillar into a life cycle sustainability assessment and presents three alternative animal welfare indicators.

**Methods:**

Animal welfare is assessed during farm life and during slaughter. The indicators differ in how they value premature death. All three consider (1) the life quality of an animal such as space allowance, (2) the slaughter age either as life duration or life fraction, and (3) the number of animals affected for providing a product unit, e.g. 1 Mcal. One of the indicators additionally takes into account a moral value denoting their intelligence and self-awareness. The framework allows for comparisons across studies and products and for applications at large spatial scales. To illustrate the framework, eight products were analysed and compared: beef, pork, poultry, milk, eggs, salmon, shrimps, and, as a novel protein source, insects.

**Results and discussion:**

Insects are granted to live longer fractions of their normal life spans, and their life quality is less compromised due to a lower assumed sentience. Still, they perform worst according to all three indicators, as their small body sizes only yield low product quantities. Therefore, we discourage from eating insects. In contrast, milk is the product that reduces animal welfare the least according to two of the three indicators and it performs relatively better than other animal products in most categories. The difference in animal welfare is mostly larger for different animal products than for different production systems of the same product. This implies that, besides less consumption of animal-based products, a shift to other animal products can significantly improve animal welfare.

**Conclusions:**

While the animal welfare assessment is simplified, it allows for a direct integration into life cycle sustainability assessment. There is a trade-off between applicability and indicator complexity, but even a simple estimate of animal welfare is much better than ignoring the issue, as is the common practice in life cycle sustainability assessments. Future research should be directed towards elaborating the life quality criterion and extending the product coverage.

**Electronic supplementary material:**

The online version of this article (10.1007/s11367-017-1420-x) contains supplementary material, which is available to authorized users.

## Introduction

Life cycle assessment (LCA) is a decision support tool to evaluate the environmental impacts of a product or service throughout its life cycle (Hellweg and Milà i Canals 2014). There is a growing interest by producers and consumers to broaden LCA to the economic and social dimensions of sustainability. It is demanded in general (Hellweg and Milà i Canals 2014) and specifically in the food sector (Nemecek et al. 2016). Although methods for life cycle costing (Swarr et al. 2011) and databases for social impact assessment (Benoit-Norris et al. 2012) exist, they are rarely applied. With the increasing consumption of animal products (Kearney 2010), also animal welfare becomes more and more relevant for sustainability assessments of food, but is even more neglected.

Animal welfare refers to the physical and mental well-being of non-human animals (Carenzi and Verga 2009). In this respect, the Farm Animal Welfare Council defined five freedoms that need to be provided to achieve animal welfare: (1) freedom from thirst, hunger and malnutrition; (2) freedom from discomfort; (3) freedom from pain, injury and disease; (4) freedom to express normal behaviour; and (5) freedom from fear and distress (Farm Animal Welfare Council 1993). Since the emotional state of animals cannot be directly measured (Carenzi and Verga 2009; Chan 2011), welfare is in practice assessed based on the satisfaction of needs. Bartussek, for example, developed an animal needs index for pigs (Bartussek 1995b), laying hens (Bartussek 1995a) and cattle (Bartussek 1996) distinguishing 30 to 38 criteria grouped into five areas of influence: (1) possibility of movement, (2) social contact, (3) floor condition, (4) climate, and (5) care intensity. He assigns scores for defined intervals of each criterion, which results in a qualitative assessment of animal welfare.

A search in Web of Science of scientific articles with the terms ‘(“life cycle assessment” OR “life cycle sustainability assessment” OR “LCA” OR “LCSA”) AND (“animal product*” OR “meat” OR “milk” OR “egg*” OR “fish*” OR “seafood” OR “diet*”)’ in their title, abstract, and keywords resulted in 1117 publications, whereas the same search with the additional term “animal welfare” resulted in 20 publications out of which only nine actually assessed animal welfare (< 1%). Seven articles assessed animal welfare for a single product type, mostly dairy products (van Asselt et al. 2015; Meul et al. 2012; Mueller-Lindenlauf et al. 2010; Del Prado et al. 2011; Schmitt et al. 2016; Zucali et al. 2016), and once poultry (Castellini et al. 2012). Two articles examined multiple products, including meat, eggs, and dairy products (Head et al. 2014; Röös et al. 2014). Each study uses different criteria to assess animal welfare. Most rely on qualitative and relative scores that do not allow for comparisons across studies. More complex approaches like the animal needs index mentioned above (van Asselt et al. 2015) or approaches that require farm visits and laboratory measurements of blood samples (Castellini et al. 2012) are not applicable at large scales. The two studies that assessed multiple product types aimed to provide a communication tool that guides consumers. However, their approaches are very generic. They either use a traffic light system for qualitative scores based on information from literature (Röös et al. 2014) or even rely on expert opinions to assign scores (Head et al. 2014). These scores are subjective and not related to a functional unit, as required for life cycle assessment. Only one study considers slaughter conditions (Röös et al. 2014) and one study considers the slaughter age (Schmitt et al. 2016) in their assessments.

Previous approaches only assessed animal welfare of livestock and rarely of fish, but ignored animal welfare of other aquatic animals and of insects, which are both food sources, too. While farm animals are already widely acknowledged as sentient beings (Carenzi and Verga 2009), the sentience of other species such as fishes and insects is debated. Although fishes are unlikely to experience the same complexity of emotions as human beings, still several studies suggest that there is anatomical, physiological, and behavioural evidence for fish sentience (Ashley 2007; Hastein et al. 2005). The increasing demand for proteins suggests that also alternative protein sources to traditional animal products will have to be exploited more in the future, and insects represent such a potential novel protein source (Boland et al. 2013; Rumpold and Schlüter 2013). Although more uncertain, research suggests that invertebrates (including insects) are sentient, in which case their welfare should be accounted for (Chan 2011; Elwood 2011).

Overall, this study aims at providing a quantitative framework for animal welfare assessment compatible with life cycle assessment. While we acknowledge that wild animals can accidentally be killed for crop and feed cultivation as well as livestock farming (see discussion), the scope of this study is limited to animals that are intentionally killed for human food consumption. It is applied to various animal-based food products including aquatic animals and insects. Finally, the impacts on animal welfare are compared for various diets.

## Methods

### Framework

Besides the intensity of distress, the duration (Morton and Hau 2010) and the number of affected animals (Chan 2011) should be taken into account in animal welfare assessments. The latter especially applies when comparing diverse animal products. Then, the welfare of invertebrates, whose sentience and consciousness is more uncertain, might gain in importance due to the large number of affected animals. At the same time, the number of affected animals allows to relate the impacts to a functional unit, such as 1 Mcal of the food product. Furthermore, the assessment should go beyond the farm gate and include the slaughter conditions to complete the life cycle. Finally, a quantitative assessment in absolute terms is preferred, as it allows for comparisons across studies. In summary, we recommend that the assessment:

1. considers the quality of an animal’s life, the lifetime, and the number of animals affected,
2. considers the conditions during farm life and slaughter (including transport to the slaughterhouse), and
3. is quantitative and related to a functional unit.

We suggest three alternative welfare indicators meeting these requirements: animal life years suffered (ALYS), loss of animal lives (AL), and loss of morally adjusted animal lives (MAL). The indicators differ in the valuation of the time lost due to premature death (lives lost). Providing three alternatives allows to choose an indicator according to the preferred ethical view or to compare the resulting impacts of all three for sensitivity analyses.

#### Animal life years suffered (indicator 1)

*Indicator 1* disregards the premature death of the animals, because, for animals living a life full of suffering, death might even mean a salvation from that suffering. The duration of suffering is then the focus and welfare loss is expressed as animal life years suffered (ALYS):

$$ \mathrm{Animal}\  \mathrm{welfare}\  \mathrm{loss}=\mathrm{number}\  \mathrm{affected}\times \left(\left[\mathrm{life}\  \mathrm{duration}\hbox{--} \mathrm{slaughter}\  \mathrm{duration}\right]\times \left[1\hbox{--} \mathrm{life}\  \mathrm{quality}\right]+\mathrm{slaughter}\  \mathrm{duration}\right) $$

#### Loss of animal lives (indicator 2)

Besides possible proximate interests such as avoiding pain, an animal ultimately strives for survival and reproduction, although not all animals might be conscious of that goal (Chan 2011). Therefore, it can be argued that not only the animal’s life quality should be considered in animal welfare assessment, but also their years of life lost. This is in line with the assessment of life cycle impacts on human health, which are typically expressed as disability-adjusted life years (DALYs), a disease burden indicator developed by the World Health Organization (Murray 1994). DALYs are composed of (1) years lost due to disability, which corresponds to the frustration of animal needs assessed in previous animal welfare studies and (2) years of life lost due to premature mortality, which is also against animals’ interest. Since animal species differ in life expectancies, we consider life fractions instead of an absolute duration. Consequently, welfare loss in *indicator 2* is expressed in number of animal lives (AL) lost:

$$ \mathrm{Animal}\  \mathrm{welfare}\  \mathrm{loss}=\mathrm{lives}\  \mathrm{lost}\ \left(\mathrm{LL}\right)+\mathrm{lives}\  \mathrm{with}\  \mathrm{disability}\ \left(\mathrm{LD}\right) $$

where

$$ \mathrm{Lives}\  \mathrm{lost}\ \left(\mathrm{LL}\right)=\mathrm{number}\  \mathrm{affected}\times \left(1\hbox{--} \mathrm{life}\  \mathrm{fraction}\right) $$

and

$$ \mathrm{Lives}\  \mathrm{with}\  \mathrm{disability}\ \left(\mathrm{LD}\right)=\mathrm{number}\  \mathrm{affected}\times \left(\left[\mathrm{life}\  \mathrm{fraction}\hbox{--} \mathrm{slaughter}\  \mathrm{fraction}\right]\times \left[1\hbox{--} \mathrm{life}\  \mathrm{quality}\right]+\mathrm{slaughter}\  \mathrm{fraction}\right) $$

In contrast to *indicator 1*, a shorter life is considered worse in this case. This was also assumed by Schmitt et al. (2016). Indicator *2* also implies that the lives of all animals are equally valued. This can be justified by assuming that all the analysed animals are sentient to some extent, and that all sentient beings have an interest in continuing to live (Francione 2010).

#### Loss of morally adjusted animal lives (indicator 3)

Alternatively, the lives of different animal species can be valued gradually, depending on their degree of self-awareness and their sense of time. Therefore, we introduce a moral value to *indicator 3* and express welfare loss as morally adjusted animal lives (MAL):

$$ \mathrm{Lives}\  \mathrm{lost}\ \left(\mathrm{LL}\right)=\mathrm{number}\  \mathrm{affected}\times \left(1\hbox{--} \mathrm{life}\  \mathrm{fraction}\right)\times \mathrm{moral}\  \mathrm{value} $$

We do not introduce the same moral value for lives with disability, because the sentience of animals is considered in the life quality. Thus, lives with disability are estimated in the same way as in indicator 2.

### Criterion 1: life quality

Animals have many needs and the frustration of any of them can lead to a loss in animal welfare. Since information about the satisfaction of all those needs is scarce, not all can be considered. While we recommend covering several needs in future studies, we select here only one criterion for each animal product as a proxy for overall life quality. Focussing on criteria with the highest availability allows to apply the indicator at large scales and facilitates comparisons across studies. The score for life quality ranges from a minimum quality of 0 to a maximum quality of 1.

For dairy cattle, life quality is approximated by the number of days per year on pasture. According to the Welfare Quality® assessment protocol for cattle (Welfare Quality® 2009a), first an index *I* is calculated:

$$ I=100\times \mathrm{days}\ \mathrm{on}\ \mathrm{pasture}/365 $$

This index is then transformed to a quality score using spline functions. If *I* is lower or equal to 50:

$$ \mathrm{Quality}=\left(1.7756\times I-0.00093197\times {I}^2-0.00010556\times {I}^3\right)/100 $$

Otherwise,

$$ \mathrm{Quality}=\left(-37.324+4.0151\times I-0.045721\times {I}^2+0.00019303\times {I}^3\right)/100 $$

For beef cattle, life quality is also approximated by the number of days per year on pasture, using the same index *I* as for dairy cattle. Two cases are distinguished for transforming the index to a quality score, depending on access to pasture before fattening. If the cattle had no access to pasture prior to fattening and *I* is lower or equal to 10:

$$ \mathrm{Quality}=\left(4.0025\times I-0.28112\times {I}^2+0.0092976\times {I}^3\right)/100 $$

Otherwise without access to pasture:

$$ \mathrm{Quality}=\left(9.3096+1.2096\times I-0.0018293\times {I}^2-0.000011980\times {I}^3\right)/100 $$

If the cattle had access to pasture prior to fattening and *I* is lower or equal to 10:

$$ \mathrm{Quality}=\left(3.9875\times I-0.22139\times {I}^2+0.0068822\times {I}^3\right)/100 $$

Otherwise,

$$ \mathrm{Quality}=\left(6.8136+1.9435\times I-0.016979\times {I}^2+0.000068633\times {I}^3\right)/100 $$

If the conditions before fattening are unknown, we suggest to take the average score of both cases.

For pigs, life quality is approximated by the surface area available for each animal (m^2^/100 kg). According to the Welfare Quality® assessment protocol for pigs (Welfare Quality® 2009b), the index *I* is calculated as:

$$ I=\left(10.3\times \mathrm{surface}\  \mathrm{area}\right)-3.09 $$

If *I* is lower or equal to 20,

$$ \mathrm{Quality}=\left(12.306\times I-0.58370\times {I}^2+0.0096231\times {I}^3\right)/100 $$

Otherwise,

$$ \mathrm{Quality}=\left(76.822+0.78238\times I-0.0075336\times {I}^2+0.000020276\times {I}^3\right)/100 $$

For broilers and laying hens, life quality is approximated by the stocking density (kg/m^2^). According to the Welfare Quality® assessment protocol for poultry (Welfare Quality® 2009c), the index *I* is calculated as:

$$ I=2.5\times \left(44-\mathrm{stocking}\  \mathrm{density}\right) $$

If *I* is lower or equal to 30:

$$ \mathrm{Quality}=\left(2.6077\times \mathrm{I}-0.051672\times {\mathrm{I}}^2+0.00050863\times {\mathrm{I}}^3\right)/100 $$

Otherwise,

$$ \mathrm{Quality}=\left(12.019+1.4058\times \mathrm{I}-0.011609\times {\mathrm{I}}^2+0.000063483\times {\mathrm{I}}^3\right)/100 $$

While the assessment protocol (Welfare Quality® 2009c) distinguishes between broilers and laying hens and does not yet provide scores for the latter, we use the same approach for laying hens as for broilers.

For the above five animal products, as the animal products most commonly consumed in Western societies, we also test alternative life quality scores (see Electronic Supplementary Material).

For Atlantic salmon, life quality is approximated by the stocking density (kg/m^3^). We define two boundary values, which are obtained from Turnbull et al. (2005). Outside of this range, animal welfare is not affected anymore because maximum or minimum welfare are already reached. Within the range, we fit a linear regression line from a minimum quality of 0 to a maximum quality of 1:

$$ \mathrm{Quality}=4.67-0.17\times \mathrm{stocking}\  \mathrm{density} $$

For shrimps, we assumed maximum life quality of 1, as, in our case studies, they were not farmed but wild-caught and, consequently, their life quality is not affected by human interference until their premature death.

For insects, we assumed a life quality of 0.999. On the one hand, insect rearing is not regulated (de Goede et al. 2013) and, as a result, insect treatment is likely to be much more inhumane than for livestock. We assume a twice as bad treatment as for chickens (e.g. half the space allowance in proportion to their body size). On the other hand, insects have a roughly 2000 times lower sentience (and moral value, see criterion 4) than chickens as the livestock closest in size and sentience. Therefore, their life quality is less affected. The maximum suffering, which is 1 for chickens, would then be 2/2000 = 0.001 for insects, and life quality is then 0.999.

Slaughter of the animals—including associated operations such as transport of livestock to the slaughterhouse—are assigned a life quality of 0, independent of the animal and production system. Stunning before slaughter is not always practiced due to religious beliefs; when it is practiced, it is not always successful at first attempt (Grandin 2010); and even if it is successful, animals are exposed to several other factors that cause immense stress during the pre-slaughter period (Terlouw et al. 2008). What differs in our assessment is the duration of suffering.

### Criterion 2: number affected

The number of animals affected per functional unit (e.g. 1 kg of meat) depends on the yield:

$$ \mathrm{Number}\  \mathrm{affected}=1/\mathrm{yield}=1/\left(\mathrm{live}\  \mathrm{weight}\times \mathrm{product}\  \mathrm{fraction}\right) $$

In case of meat or fish (as opposed to milk and eggs), the live or slaughter weight is usually specified and has to be converted to the product weight that is ready to be cooked and eaten with the product fractions given in Table 1. For shrimps and insects, we assume a product fraction of 100%. Further allocation of different meat qualities can be done in subsequent modelling.

**Table 1:** Product fractions

Product	Live weight	Slaughter weight	Reference
Cattle (beef)	0.353	0.679	(Alig et al. 2012)
Pigs	0.417	0.528	(Alig et al. 2012)
Chickens (broilers)	0.699	0.724	(Haslinger et al. 2007)
Atlantic salmons	0.560	0.620	(Bencze Rørå et al. 1998)

More than one animal can be affected by a product, such as male chicks in egg production and bobby calves in milk production. Moreover, when catching shrimps, other species are accidentally caught of which some are discarded and not used as food. Such additional life loss is also attributed to the respective product and, as such, reduces the product yield per animal killed. However, we disregarded the possible use of animal products such as fishmeal and fish oil as animal feed (Shepherd and Jackson 2013).

Contrariwise, one animal can produce more than one product. This was accounted for by converting the by-products to equivalents of the main product, and this increases the yield per animal. For beef cattle, a monetary value fraction of 0.83 was assumed for the meat, while other profit can be made from edible offal, semen, and leather (Mekonnen and Hoekstra 2010). We assumed dairy cows to weigh 500 kg at the end of their life by selecting a low value of reported live weights of beef cattle. Bobby calves were assumed to have a slaughter weight of 17 kg (Flysjö et al. 2011). We used a ratio of 11:1 as price ratio per kg between meat and milk (More O’Ferrall 1982). For eggs, value fractions were derived from the revenue of eggs compared to that of meat from spent hens which are provided for different egg production systems (Dekker et al. 2011). When catching shrimps, some of the accidentally caught other species are not discarded but used as additional food (by-catch) (Ziegler et al. 2011). We assumed equal values per mass for shrimps and by-catch.

Since calories better represent the function of food than mass does, we convert the number of affected animals per kg to the number of affected animals per Mcal (1000 food calories). The caloric contents are displayed in Table 2.

**Table 2:** Caloric content of animal products

Product	Calories (kcal/kg)	Reference
Beef	2760	(USDA 2016) Code 13330
Pork	2630	(USDA 2016) Code 10219
Poultry	2150	(USDA 2016) Code 05006
Eggs	1430	(USDA 2016) Code 01123
Milk	610	(USDA 2016) Code 01211
Salmon	2080	(USDA 2016) Code 15236
Shrimp	850	(USDA 2016) Code 15270
Cricket	1200	(Huis et al. 2013)
Mealworm	2060	(Huis et al. 2013)

### Criterion 3: time

The slaughter age defines the life duration, while the life fraction is derived from the ratio of slaughter age to life expectancy:

$$ \mathrm{Life}\  \mathrm{fraction}=\mathrm{slaughter}\ \mathrm{age}/\mathrm{life}\  \mathrm{expectancy} $$

Life spans and life expectancies are compiled in Table 3. Life span is here defined as the maximum number of years an animal of that species can live, while life expectancy is the average number of years that an animal is expected to live at birth. Premature death can also happen in wildlife, for example, caused by natural predators such as wolves, pumas, and leopards. This reduces the life expectancy compared to the life span. Such differences can be substantial: for example, buffalos (wild relatives of domestic cattle) have a natural life span of 20–23 years, but live, on average, for only 4.3 to 6.3 years; and warthogs (wild relatives of domestic pigs) live, on average, less than 3 years despite a natural life span of 17 years (Spinage 1972). Although farmed animals can be victims of predators as well (Treves and Karanth 2003), captive animals often have higher life expectancies than free-living animals (Mason 2010) if they would not be slaughtered. As a conservative approach, we still assume the life expectancy of an animal in wildlife as the reference for our analysis, however, without granting benefits when a farm animal lives longer (i.e. the life fraction is limited to 1), which can happen for dairy cows in some systems.

**Table 3:** Demographic characteristics of animals

Animal	Life span (years)	Life expectancy (years)	Reference
Cattle (beef and dairy cattle)	20	5^a^	(Delgado et al. 2017, Spinage 1972)
Pigs	15	3^a^	(Delgado et al. 2017, Spinage 1972)
Chickens (broilers and laying hens)	7.5 (5–10)	3	(Delgado et al. 2017, Komiyama et al. 2004)
Atlantic salmons	13	6	(Kalman and Sjonger 2007)
Southern pink shrimps		1.67	(García-Isarch et al. 2013)
Mealworm (*Tenebrio molitor*)		0.5	(Tran et al. 2016)
House cricket (*Acheta domesticus*)		0.21	(Walker 2014)

^a^Life expectancy of wild relative

If more than one animal is affected by a product, the weighted average of slaughter ages is taken. This concerns egg and milk production. Male chicks are culled at the age of 1 day because they cannot lay eggs, while laying hens are slaughtered after more than 1 year (Aerts et al. 2009). Similarly, in some milk production systems, bobby calves are slaughtered a few days after being born because not all calves can be raised for economic profit, whereas the dairy cow lives for a few years (Flysjö et al. 2011).

The time from catching the animals to be slaughtered until they actually die is described here as the slaughter duration, while slaughter fraction is relative to the life span. For livestock, it includes the loading of animals into a transport vehicle, the journey to the slaughterhouse, and the waiting in the slaughterhouse. The FAO recommends that cattle should not be transported for longer than 36 h (Chambers et al. 2001), and the EU limits the transport of poultry without water to 12 h (Eyes on Animals 2013). Loading the truck beforehand and waiting for slaughter afterwards can each take another couple of hours (Eyes on Animals 2013). Based on that, we assumed a slaughter duration of 1 day for livestock. In extreme cases, transport can even take a month (Independent 2008). Wild aquatic animals usually die from suffocation, which takes some minutes. The operation before that, for example trawling with a net, can take several hours (Braithwaite 2010). Only shrimps are concerned by wild catch in our study, and we assumed a slaughter duration of 1 h. In aquaculture, in our case for salmon production, we assumed fish is killed by a gill cut or by stunning with carbon dioxide followed by a gill cut, which takes, on average, 5–6 min until brain function is lost (van de Vis et al. 2003). Adding time for pre-slaughter management, we assumed a slaughter duration of 10 min. In contrast, insects are often killed by freezing, and we assumed that it takes 10 min (Roscoe et al. 2016), although it can even take 1 h (Lo Pinto et al. 2013) (Table 4).

**Table 4:** Slaughter duration

Livestock	Fisheries	Aquaculture	Insects
1 day	1 h	10 min	10 min

### Criterion 4: moral value

Scientific evidence shows that pigs, cattle, and some birds are self-aware and plan for the future. Also chickens recognise individuals and plan at least for the near future (Marino 2017). Fishes are less likely to be self-aware, but demonstrated their ability of remembering the past by still remembering a hole in a net after almost a year (Singer 2011). Insects are much less understood and might not be self-aware. However, it would be misleading to draw conclusions from their brain size about their intelligence. Social insects such as ants and bees were found to be much more intelligent than previously thought (Chittka and Niven 2009). Although intelligence is not a measure of self-awareness, we give them the benefit of the doubt (Singer 2011) and assume that they are to some extent self-aware. Still, the degree of self-awareness and sense of time of all the animals under investigation is lower than of human beings who are the only ones with a biographical sense, who tell stories about their past and hope to achieve something in the far future (Singer 2011).

We assigned each species a moral value based on their expected intelligence relative to a human being (Table 5). We approximated intelligence either by brain mass, number of total neurons, or number of cortical neurons, depending on data availability. Among these, the number of cortical neurons seems to be the best measure of intelligence and is the only one that can explain the superior cognitive abilities of human beings. An elephant’s brain, for example, is about three times larger and contains three times more neurons than the average human brain, but only a third of the cortical neurons as found in human brains (Herculano-Houzel et al. 2014).

**Table 5:** Moral valuation of animal lives

Animal	Proxy animal	Cortical neurons	Neurons	Brain mass	Moral value
Human^a^	–	16 billion	86 billion	1508 g	1
Cattle^b^	–		3 billion		0.035
Pig^c^	–	432 million			0.027
Chicken^d^	Red junglefowl	61 million			0.0038
Salmon^e^	Shark			1.8 g	0.0012
Shrimp^f^	Lobster		100,000		1.2 × 10^−6^
Cricket^g^	Fruit fly and ant		250,000		2.9 × 10^−6^
Mealworm^g,h^	Fruit fly, ant and zebrafish		25,000		2.9 × 10^−7^

^a^(Azevedo et al. 2009, Herculano-Houzel 2009)

^b^(Herculano-Houzel 2016)

^c^The number only refers to neocortical neurons (Jelsing et al. 2006); hence, it underestimates the cortical neurons

^d^(Olkowicz et al. 2016)

^e^The body mass of a salmon was assumed to equal 4.5 kg (FRS Marine Laboratory 2006), while the brain:body mass ratio was assumed to equal that of a shark—1:2496 (Serendip 2016)

^f^(Lobster Institute 2016)

^g^(Burne et al. 2011; Shulman and Bostrom 2012)

^h^The factor difference between an adult insect and the larva (a mealworm is the larva of the mealworm beetle) was assumed to equal that of a zebrafish, which is a factor of 10: a larval zebrafish has 100,000 neurons (Naumann et al. 2010), while an adult zebrafish has 1 million neurons (Alivisatos et al. 2012)

### Case study

To illustrate the application of the framework, we perform a case study comparing different types of animal products and different diets. The data for the evaluation are obtained from a literature review. Most information is retrieved from LCA studies on environmental aspects of animal production, which provide parameters relevant for an animal welfare assessment. In total, our database covers 50 cases for eight animal products.

The sensitivity of animal welfare to changes in the parameters describing the criteria was tested. Individual input parameters were halved and the effect on animal welfare (output) was quantified using (MacLeod et al. 2002):

$$ S=\frac{\Delta  \mathrm{Output}/\mathrm{output}}{\Delta  \mathrm{Input}/\mathrm{input}} $$

We estimated animal welfare for the world average per capita consumption of animal products in the year 2011 based on data from FAOSTAT (FAO 2015). The consumption of beef, pig, and chicken meat was scaled up to compensate for the neglected consumption of mutton, goat, and other meat. Although it is a large simplification, all fish consumption was represented by salmon and all seafood consumption by shrimps. A hypothetical diet without seafood, a diet without birds (poultry and eggs) and an ovo-lacto-vegetarian diet were constructed by replacing all missing animal proteins by proteins from the remaining animal products. An additional vegetarian diet was constructed by assuming that missing animal proteins are substituted by proteins from plants, without the need to increase the consumption of milk and eggs (Table 6).

**Table 6:** Animal product composition in kg/(a × capita) of different diets with equal protein intake

Product	Omnivore incl. seafood	Omnivore	Omnivore without birds	Veg. (animal substit.)	Veg. (plant substit.)
Beef	10.1	12.7	18.7	–	–
Pork	16.7	21.0	31.0	–	–
Poultry	15.6	19.6	–	–	–
Milk	90.7	114	168	259	90.7
Eggs	8.95	11.2	–	25.6	8.95
Salmon	14.2	–	–	–	–
Shrimps	4.93	–	–	–	–

Following the LCA framework, the life cycle inventory—the diets—was multiplied with the impact characterisation factor—the developed welfare indicator—to yield the total impact on animal welfare caused by the respective diet.

## Results

### Animal product comparison

**Fig. 1:**
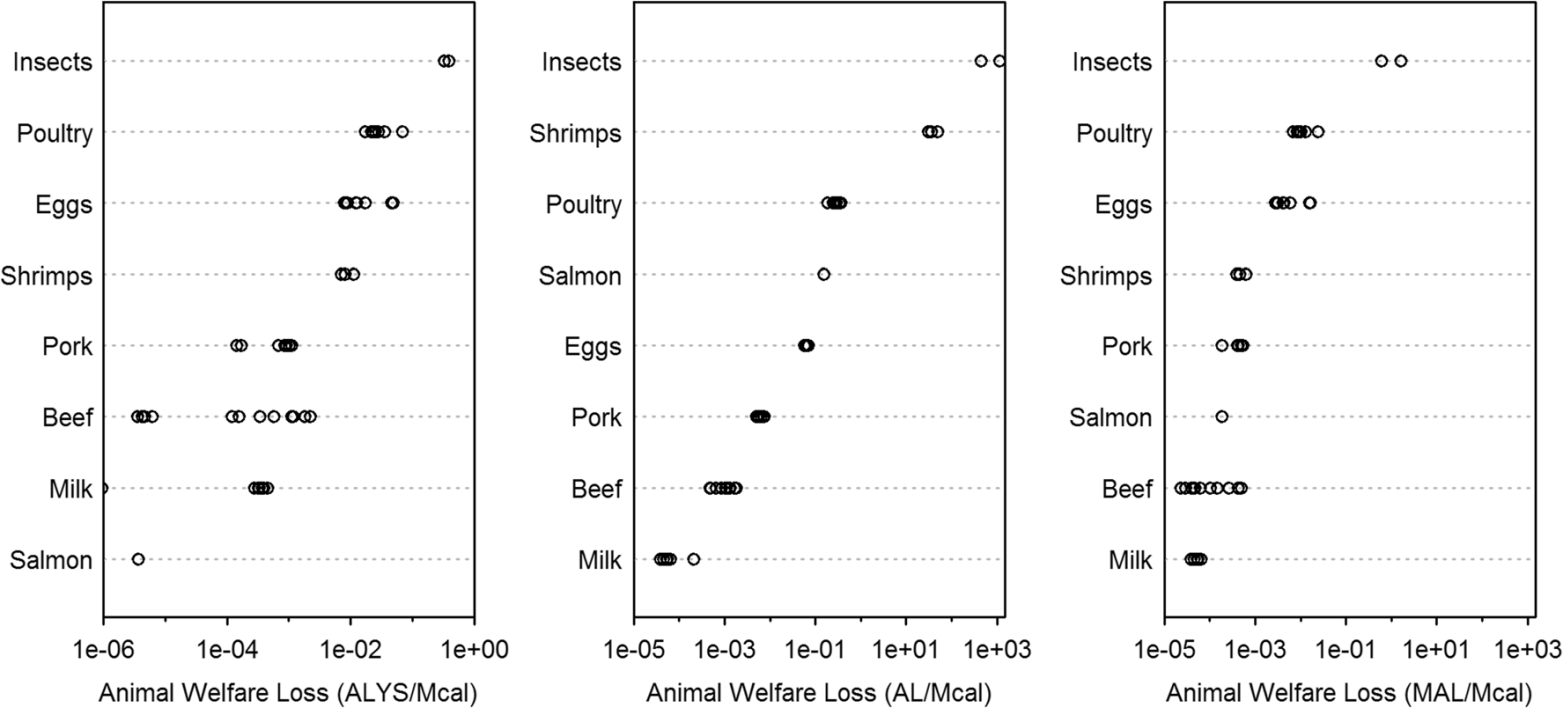
Animal welfare loss of various food products using three alternative indicators. Different estimates (circles) for the same animal product represent different production systems and/or countries

There are large differences in animal welfare loss per Mcal by food products (Fig. 1, Table 7), ranging from 3.6 × 10^−6^ ALYS for the average salmon to 0.36 ALYS for the average insect (indicator 1), from 7.0 × 10^−5^ AL for the average milk to 778 AL for the average insect (indicator 2), and from 4.5 × 10^−5^ MAL for the average milk to 1.1 MAL for the average insect (indicator 3). Taking a concrete example, consuming 1 Mcal of poultry is, depending on the indicator, 29 to 47 times worse than consuming the same amount of pork. In contrast, the worst poultry causes only twice to four times as much welfare loss as the best poultry from our set of case studies. This highlights that it is more decisive which animal product we consume than in which production system the animal is raised. Still, there can be considerable differences among production systems with regards to ALYS. According to indicator 1, US beef from conventional agriculture is over 200 times worse than when raised entirely on pasture (see Electronic Supplementary Material).

**Table 7:** Average animal welfare evaluation of various food products

Product	Life quality (−)	Life fraction (−)	Life duration (years)	Number affected (−)	Moral value (−)
Insects^a^	0.999	*0.59*	*0.21*	**2720**	*1.6* × *10* ^*−6*^
Shrimps^b^	*1.0*	0.50	0.83	**66**	*1.2* × *10* ^*−6*^
Poultry^c^	**0.39**	**0.060**	*0.18*	0.63	0.0038
Salmon^d^	*1.0*	0.21	1.3	0.40	0.0012
Eggs^e^	**0.60**	0.24	0.71	0.10	0.0038
Pork^f^	0.80	**0.16**	0.48	0.018	0.027
Beef^g^	0.66	0.31	**1.6**	*0.0039*	**0.035**
Milk^h^	0.76	*0.93*	**6.3**	*0.00013*	**0.035**
CV	0.29	0.76	1.4	2.8	1.4
*S* (indic. 1)	− 2.2	–	1.0	1.0	–
*S* (indic. 2)	− 0.32	− 0.23	–	1.0	–
*S* (indic. 3)	− 2.0	0.89	–	1.0	0.069

The two worst performing products with regards to the criteria underlying the indicators are presented in bold, while the two best performing products are presented in italic. CV is the coefficient of variation between the eight product averages. The last three rows indicate the sensitivity (*S*) of the respective animal welfare indicators to changes in any of the criteria

^a^(Hanboonsong et al. 2013; Tran et al. 2016; Finke 2002)

^b^(Ziegler et al. 2011)

^c^(Alig et al. 2012; Castellini et al. 2006; Boggia et al. 2010)

^d^(Johansson et al. 2006; Bergheim et al. 2009; FRS Marine Laboratory 2006)

^e^(Dekker et al. 2011, Leinonen et al. 2012)

^f^(Basset-Mens and van der Werf 2005; Pelletier et al. 2010a; Honeyman 2005; Honeyman et al. 2006)

^g^(Ridoutt et al. 2012; Nguyen et al. 2010; Pelletier et al. 2010b)

^h^(Flysjö et al. 2011; Hietala et al. 2015)

Although insects live for a longer life fraction until they are slaughtered and their life quality is assumed to be less compromised because they are less sentient than birds and mammals, the overall welfare loss is still immense because their small body sizes require a large number of insects to satisfy a certain demand for animal food products. In contrast, milk products perform relatively well in the categories life quality and life fraction, and perform best in terms of number of animals affected due to the large milk yield per cow, which results in lower animal welfare loss than other products. Similarly to insects, shrimps are small and, due to the large number of shrimps affected, the overall animal welfare loss is very large (indicator 2), unless their moral value is considerably downgraded (indicator 3) or the shortening of their life is not considered a welfare loss (indicator 1; shrimps are wild-caught in our case studies and, therefore, the suffering is very short). Salmon performs best in terms of ALYS, because the stocking density in our study is high enough and, thus, does not reduce life quality and the slaughter is assumed to be rapid. Among the three commonly eaten meat products in Western societies, poultry causes the highest animal welfare loss, followed by pork and finally beef. If someone wants to reduce animal suffering, but the step to a vegan diet seems too demanding, it is, therefore, recommended to stop eating poultry before pork or beef. This dietary change is also endorsed by other scientists as a first step (Matheny 2003; Lamey 2007).

The difference in animal welfare can be explained to a large extent by the number of animals affected. It is the parameter with the largest coefficient of variation among the products, and all indicators are highly sensitive to a change in this parameter (Table 7). In contrast, animal welfare is hardly sensitive to the life quality and the time component (life fraction or life duration) in indicator 2, but they gain importance in indicators 1 and 3.

### Diet comparison

**Fig. 2:**
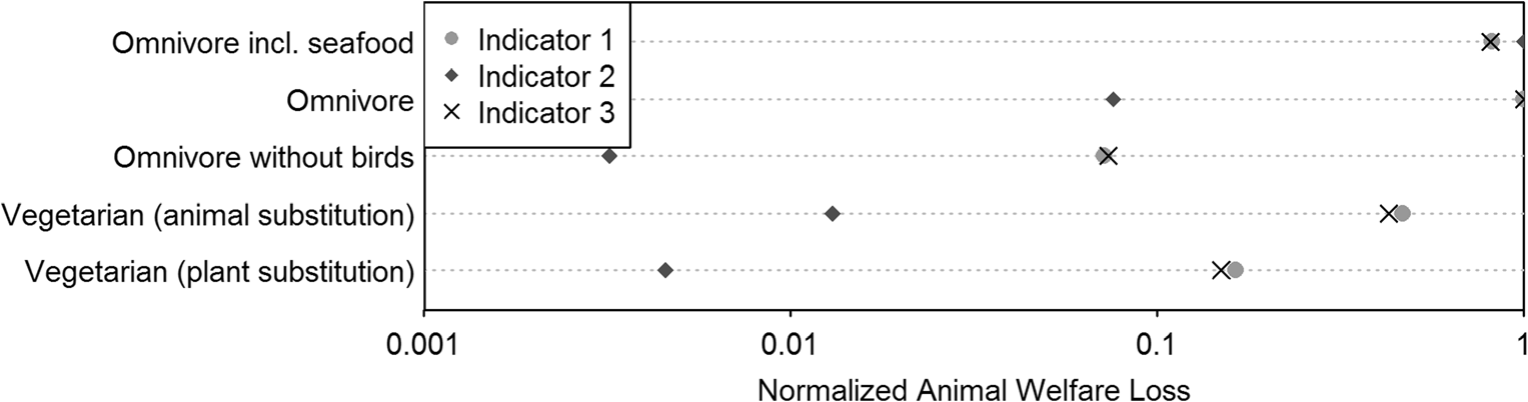
Animal welfare loss of various diets**.** The diets correspond to those in Table 6

The world average per capita consumption of animal products in a year has large impacts on animal welfare, with an average estimate of 177 AL (indicator 2, Fig. 2). The large welfare loss is mainly driven by the consumption of shrimps which stick out due to their small body sizes. As a result, a diet without seafood causes much less impacts, with an average estimate of 13 AL. When using any of the other two indicators, the ranking changes but the difference between these two diets is small. A vegetarian diet further improves animal welfare, with an average estimate of 2.3 or 0.8 AL (indicator 2), depending on how the missing animal proteins are substituted. This welfare loss is mainly due to the consumption of eggs. Eggs cause less suffering than poultry, but still perform worse than pork and beef according to all three indicators (Fig. 1). That is also why a diet including pork, beef, and milk, but excluding poultry and eggs is better in terms of animal welfare than an ovo-lacto-vegetarian diet for all three indicators (Fig. 2).

## Discussion

### Animal welfare assessment

While this paper presents a first generic quantitative assessment of animal welfare in the context of life cycle sustainability assessment, the evaluation of life quality is very simplified and should be elaborated in future assessments. It is restricted to space allowance and freedom of movement, whereas many more conditions determine an animal’s welfare. The developed indicator does neither account for all the five freedoms mentioned in the introduction nor for especially cruel treatments such as highly accelerated growth rates of broiler chicken, impeding walking (Bessei 2006), or dehorning, branding, and castration even of extensively held cattle without anaesthesia (Petherick 2005), not to mention the slaughter. It has to be reemphasized that the simplified life quality indicator hides the fact that also extensively held animals suffer from inhumane treatments, as the example of cattle demonstrates. A more comprehensive life quality indicator like the one developed by Bartussek (Bartussek 1995a, 1995b, 1996) and the Welfare Quality® Consortium (Welfare Quality® 2009a, 2009b, 2009c) can, however, not be applied at large scale due to data scarcity. Therefore, a balance must be found between scalability and indicator complexity.

Previous indicators, such as those by Bartussek (1996, Bartussek 1995a, 1995b) and the Welfare Quality® Consortium (Welfare Quality® 2009a, 2009b, 2009c), neglected the temporal aspect and the number of animals affected. We consider these aspects as crucial for assessing animal welfare. Furthermore, it is consistent with the assessment of human health impacts in LCA where both aspects are also included. The three indicators developed in this study differ especially with regards to the valuation of lives lost due to premature death. It only affected the ranking of salmon and shrimps, whereas other product rankings remained consistent. This demonstrates the robustness of the main results: insects perform worst and poultry causes more animal welfare loss than pork and beef.

It has to be noted that the average estimates per product are based on a limited number of cases, ranging from 2 for salmon up to 12 for beef, and that the unweighted average is unlikely to represent the actual shares of countries and farming systems (such as extensive versus intensive [see e.g. beef in Fig. 1] or aquaculture versus wild fisheries). Also the number of products is still limited in this study. It would be valuable to also assess the animal welfare loss of meat and milk from muttons and goats. Products derived from milk might perform worse than milk itself, although still better than the other investigated products. As an example, 1 kg of cheese requires about 7 kg of milk (Scherer and Pfister 2016), but also contains about six times as many calories (USDA 2016). Especially seafood is poorly represented in our study. Since fishes vary greatly in sizes, salmon is not necessarily representative for other fish products. For instance, Atlantic herring, another commonly eaten fish, typically weighs < 1 kg (Binohlan and Bailly 2016) as opposed to 4.5 kg assumed for salmon in this study, which leads to a higher number of animals affected.

### Animal welfare in crop cultivation

Crop cultivation, when intensive, is not free from causing harm to animals either (Davis 2003). It is less obvious because the animals are usually not harmed intentionally, with the exception of applying pesticides. Still, farm machinery like ploughs and harvesters can accidentally kill wild animals living on agricultural fields, from insects to small rodents. It is debated whether products from pasture-raised ruminants like cattle cause less harm than crops from intensive cultivation (Davis 2003) or not (Matheny 2003; Lamey 2007). In reality, neither all crops are cultivated intensively, nor all cattle are raised on pastures. Globally, only 27% of the cattle and buffalo population is fully raised on pastures (derived from absolute numbers in Table 2.9 of Steinfeld et al. 2006) and, besides grass, they consume crops with an average efficiency of 1.1 kg concentrate feed per 1 kg beef, compared to 4.0 kg concentrate feed per 1 kg beef from industrial systems (Mekonnen and Hoekstra 2010).

Quantifying the welfare of wild animals was beyond the scope of this study. That can be justified by the distinction between intentional and accidental killing of animals—the same distinction is made for human beings where manslaughter and murder are valued differently in most legal systems (Lamey 2007)—and it might lead to double counting of impacts on ecosystem quality quantified in LCA studies (e.g. for toxic emissions and land use). Still, we recognise the substantial consequences of accidental killing in intensive crop cultivation and the need for further research in this field.

### Trade-offs with environmental impacts

Apart from a loss in animal welfare, livestock also leads to severe environmental degradation. Most notably, it represents the largest anthropogenic land use (70% of agricultural land, Steinfeld et al. 2006) and the largest emitter of greenhouse gases (15% of the global warming effect, Gerber et al. 2013). Per kg product, beef causes the highest impacts in terms of land and energy use, global warming, acidification, and eutrophication potential, followed by pork, chicken, eggs, and milk with the exception of milk causing higher impacts than eggs in terms of acidification and eutrophication potential (de Vries and de Boer 2010). This shows important trade-offs between welfare loss and environmental degradation among the different animal products. Beef is the meat with the lowest loss in animal welfare, but the highest environmental impacts. By contrast, poultry is the meat with the highest loss in animal welfare, but the lowest environmental impacts. A synergy exists for milk which is the animal product with the lowest loss in animal welfare and the lowest environmental impacts. A comparative study on meat substitutes has shown that lab-grown meat performs considerably worse than chicken meat and dairy milk, whereas edible insects are beneficial for the environment. Soya meal-based substitutes represent the most environmentally friendly alternative (Smetana et al. 2015).

Numerous studies have shown that a diet with less animal products and especially a vegan diet benefits the environment (e.g. Jalava et al. 2014; Stehfest et al. 2009; Goldstein et al. 2016; Meier and Christen 2013). Another study has estimated that, in the Netherlands, land would be used most efficiently if 12% of dietary proteins come from animal sources, especially ruminants (e.g. dairy milk) who are raised on land unsuitable for crop production and are fed by grass, crop residues, and food waste (van Kernebeek et al. 2016). However, currently, 33% of proteins come from animal sources at the global level, with higher intakes in developed countries (Steinfeld et al. 2006). This still implies a necessary reduction in animal products by almost two thirds.

### Trade-offs with human health through nutrition

Similar to environmental impacts, there are trade-offs and synergies between welfare loss and human nutrition among the different animal products. Some food guides, like Harvard’s Healthy Eating Pyramid, recommend white meat such as chicken over red meat such as beef (Reedy and Krebs-Smith 2008), whereas chicken reduces animal welfare more. While Harvard’s guide discourages from consuming dairy products, other food guides, including US Department of Agriculture’s MyPyramid, recommend a higher intake of milk than meat (Reedy and Krebs-Smith 2008), and milk also causes less animal welfare loss. However, since about 70% of the global population have hypolactasia, which often leads to intolerance to lactose (a sugar found in dairy milk), many people should limit or avoid dairy products to prevent symptoms like abdominal pain (Lomer et al. 2008).

Diverse health concerns about the consumption of animal products have been raised. While the health of under- or malnourished people in developing countries might benefit from a higher consumption of animal products, the World Health Organization recommends a lower consumption of animal products in developed countries, as many non-communicable diseases including cardio-vascular diseases, diabetes, and certain types of cancer are associated with high intakes of animal products. The World Organization for Animal Health estimates that as much as 60% of human pathogens and 75% of novel diseases are transmitted from animals to humans during their consumption (Steinfeld et al. 2006). In Western societies, a very low meat consumption (less than weekly) also contributes to lower mortality risk and higher life expectancy as opposed to the average diet (Singh et al. 2003). Others even advise against the consumption of dairy products in a vegetarian diet—i.e. they recommend a vegan diet—to avoid the negative health effects of saturated fats and cholesterol in these and other animal products, as the beneficial nutrients contained therein can also be efficiently obtained from plant-based food (Lanou 2009).

### Barriers and opportunities to increase animal welfare

Individuals often face value conflicts about ethical food choices. In other words, contradictory priorities compete in reaching a decision, which leads to paradoxical outcomes (Szmigin et al. 2009). As citizens, they are aware of a loss in animal welfare in livestock husbandry and advocate good living conditions for farm animals. In contrast, as consumers, they suppress any cognition with the animals (Schröder and McEachern 2004) and decide based on economic and social factors (Szmigin et al. 2009). On the one hand, animal products from industrial systems are more economical than products with higher welfare standards. On the other hand, the eating habits of family and friends determine the social norms—typically an omnivore diet. As a consequence, consumers disconnect their attitude from their behaviour. In addition, food neophobia (the openness to trying novel food) prevents many consumers from trying meat substitutes as an alternative protein source (Hoek et al. 2011).

There are three alternative ways to improve animal welfare: (1) legislation can enforce higher welfare standards, (2) retailers can insist in higher welfare standards and offer more meat substitutes, and (3) consumers can demand higher welfare standards and meat substitutes within a free market. While the World Trade Organization does not allow nations to restrict imports of cheaper, less animal-friendly products, which could lead to trade substitutions and render regulations ineffective, retailers can decide to refuse selling products below a certain welfare standard and thereby have a larger influence on farming practices than regulations (Matheny and Leahy 2007). The costs to farmers for legislation can be substantial, while the costs to consumers are usually small. Therefore, consumers can express their desires for higher welfare conditions or plant-based substitutes without a significant economic disadvantage (Webster 2001), which, in turn, gives incentives to retailers to rethink their product portfolios. However, consumers are often hesitant about dietary changes and prefer to cook and eat what they are familiar with. Again, retailers can influence it and facilitate dietary changes by offering more meat substitutes and making them more attractive by improving the convenience and resemblance to meat (Hoek et al. 2011; Schösler et al. 2012).

In line with the “Three R’s”, developed as ethical guide for animal experimentation (Russell and Burch 1959), we recommend the following hierarchical principles for increasing animal welfare in food production:

1. Replace: avoid animal products in your diet and replace them by suitable plant-based products.
2. Reduce: minimise the number of animals affected by your diet. This can be done by reducing the overall consumption of animal products, or by shifting the consumption from poultry to pork and beef which have higher yields.
3. Refine: minimise animal suffering by choosing products from farms providing conditions that allow animals to satisfy their needs.

## Conclusions

This study proposes a framework for animal welfare assessment. While the assessment is simplified, it allows for a direct integration into life cycle sustainability assessment. There is a trade-off between applicability and indicator complexity, but even a simple estimate of animal welfare is much better than ignoring the issue, as is the common practice in life cycle sustainability assessments. The framework aims to enable routine assessments of animal welfare. Three alternative animal welfare indicators are suggested: (1) animal life years suffered (ALYS), (2) loss of animal lives (AL), and (3) loss of morally adjusted animal lives (MAL). The indicators all consider three criteria: (1) the life quality of an animal on the farm and during the slaughter process, (2) the slaughter age either as life duration or life fraction, and (3) the number of animals affected for providing a product unit. One of the indicators additionally takes into account a moral value assigned to animals based on the number of neurons or brain mass as a proxy for their intelligence and self-awareness. The indicators differ in the valuation of the time lost due to premature death. Providing multiple alternatives allows to choose an indicator according to the preferred ethical view or to conduct sensitivity analyses.

The indicators are applied to eight products: beef, pork, poultry, milk, eggs, salmon, shrimps, and insects. Animal welfare loss is most influenced by the number of animals affected. Consequently, the difference in animal welfare is often larger for different animal products than for different production systems of the same product. While milk reduces animal welfare the least according to most indicators and criteria, insects perform worst despite a much lower assumed sentience. The sentience as a proxy for the moral value is the most normative choice and requires special attention in future research comparing welfare across species. As a note of caution, the investigated case studies might not be representative for global production. Future research should extend the database to improve the representativeness and the product coverage. If more welfare-relevant variables are reported in the future, the life quality criterion can also be elaborated to account for further animal needs.

In Western societies with a high meat consumption, a reduced intake of animal products benefits their health, the environment, and the production animals. Still, consumers often face high personal barriers. Besides a reduction, our study shows a further opportunity to improve animal welfare: a shift to other products, usually derived from larger animals.

## Electronic supplementary material

ESM 1 (PDF 248 kb)

ESM 2 (CSV 13 kb)
