# Supplementary material for: Framework for integrating animal welfare into life cycle sustainability assessment
Source: Int J Life Cycle Assess. 2017 Nov 20;23(7):1476–90. doi: 10.1007/s11367-017-1420-x (PMC6435210; doi:10.1007/s11367-017-1420-x)
Supplement: Supplementary file 1 — (PDF 248 kb) [file 11367_2017_1420_MOESM1_ESM.pdf]

SUPPLEMENTARY MATERIAL

Framework for Integrating Animal Welfare into Life Cycle Sustainability Assessment

Laura Scherer[1*], Brian Tomasik[2], Oscar Rueda[3], Stephan Pfister[4]

[1]Institute of Environmental Sciences (CML), Leiden University, Leiden, Netherlands

[2]Foundational Research Institute, Berlin, Germany

[3]Effective Altruism Foundation, Basel, Switzerland

[4]Institute of Environmental Engineering, ETH Zurich, Zurich, Switzerland

* Institute of Environmental Sciences (CML), Leiden University, Einsteinweg 2, 2333 CC Leiden, Netherlands, email: l.a.scherer@cml.leidenuniv.nl

Methods

Criterion 1: Life quality

As a sensitivity analysis, we examine the change in the animal welfare indicators when using alternative scores for the life quality of animals used for the most commonly eaten animal products in Western societies: cattle, pigs, and poultry. We define two boundary values for each criterion. Outside of this range, animal welfare is not affected anymore because maximum or minimum welfare are already reached. Within the range, we fit a linear regression line from a minimum quality of 0 to a maximum quality of 1.

For beef and dairy cattle, life quality is approximated by the number of days per year on pasture and boundary values are obtained from Bartussek (1996):

$$\text{Quality} = 1/270 \cdot \text{Days on pasture}$$

For pigs, life quality is approximated by the surface area available for each animal (m^2/animal) and boundary values are obtained from Bartussek (1995b):

$$\text{Quality} = -0.83 + 27.78 \cdot \text{Surface area} / \text{Live weight}^{0.67}$$

For broilers and laying hens, life quality is approximated by the average of two criteria, the stocking density (animals/m^2):

$$\text{Quality} = 2.33 - 0.33 \cdot \text{Stocking density}$$

and the pasture area available for each animal (m^2/animal) with boundary values obtained from Bartussek (1995a):

$$\text{Quality} = -0.20 + 0.08 \cdot \text{Pasture area}$$

Results

Animal product comparison

When using alternative scores for life quality, the ranking among animal products hardly changes (Table S1). Poultry and eggs remain the products with the lowest life quality. However, pigs now have a lower life quality than cattle for either beef or milk. In general, the life quality is lower with the alternative scores, except for a

slight increase in life quality for beef. This can be explained by the linear relationship assumed in the alternative scores. In the default scores, life quality first improves quickly with better conditions, e.g. a higher space allowance, and then the improvement slows down. Consequently, very low values, such as for poultry, are more seldom when using the default scores. The variation among animal products is also higher when using the alternative scores.

Table S1. Average life quality evaluation of various food products. Products for which the life quality score was changed are highlighted in grey. The two worst performing products are presented in bold, while the two best performing products are presented in italic. CV is the coefficient of variation between the eight product averages.

Product	Life quality (-), default*	Life quality (-), alternative
Insects	0.999	0.999
Shrimps	*1.0*	*1.0*
Poultry	**0.39**	**0.082**
Salmon	*1.0*	*1.0*
Eggs	**0.60**	**0.21**
Pork	0.80	0.49
Beef	0.66	0.68
Milk	0.76	0.72
CV	0.29	0.56

*As in Table 7 of the manuscript.

Despite the changes in life quality, overall animal welfare of animal products only slightly changes. The change in ranking of the life quality for pork does not affect the ranking of overall animal welfare, as, also with the higher life quality assumed in the default scores, pork performs worse than beef and milk. The only ranking that changes is a switch in the ranking of pork and shrimps in indicator 3 (Fig. S1); however, the welfare scores remain close to each other.

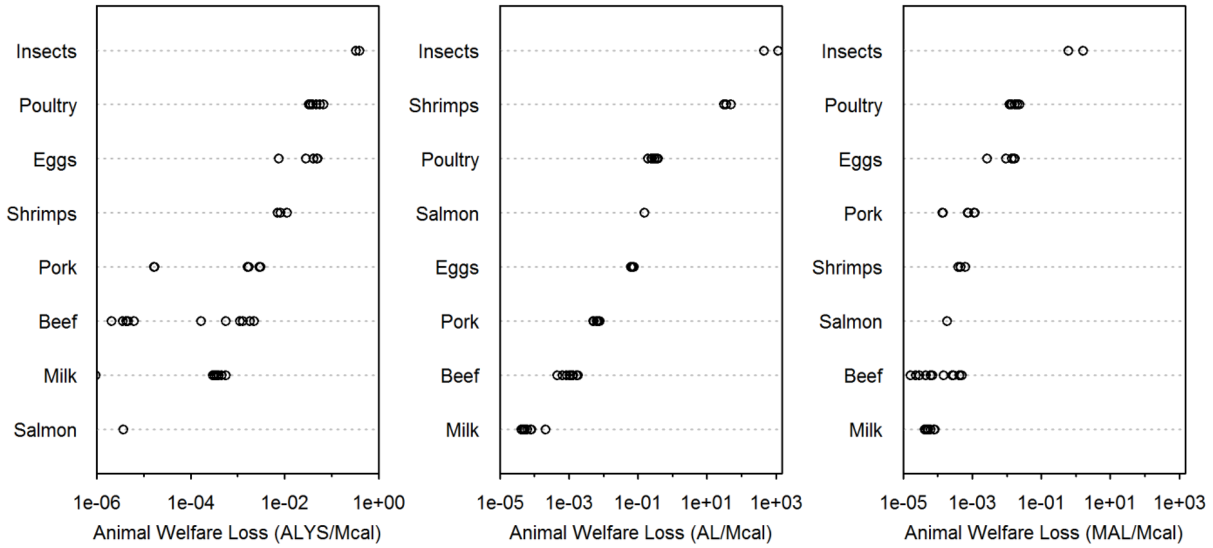

Fig. S1. Animal welfare loss of various food products using three alternative indicators. Different estimates (circles) for the same animal product represent different production systems and/or countries.

Diet comparison

The diet comparison leads to the same conclusions with the alternative life quality scores: a diet including pork, beef and milk, but excluding poultry and eggs is better in terms of animal welfare than an ovo-lacto-vegetarian diet for all three indicators (Fig. S2).

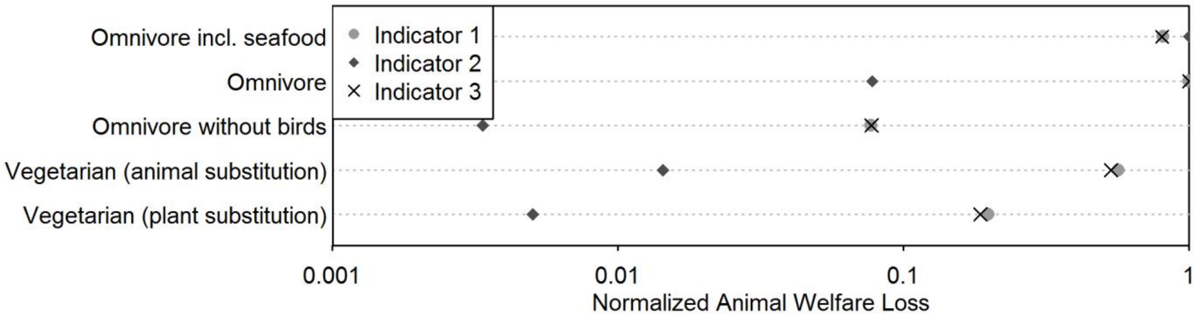

Fig. S2. Animal welfare loss of various diets. The diets correspond to those in Table 6 of the manuscript.

References

Bartussek H (1995a) Tiergerechtheitsindex für Legehennen (engl.: Animal welfare index for laying hens). Bundesanstalt für alpenländische Landwirtschaft, Irdning, Austria

Bartussek H (1995b) Tiergerechtheitsindex für Mastschweine (engl.: Animal welfare index for fattening pigs). Bundesanstalt für alpenländische Landwirtschaft, Irdning, Austria

Bartussek H (1996) Tiergerechtheitsindex für Rinder (engl.: Animal welfare index for cattle). Bundesanstalt für alpenländische Landwirtschaft, Irdning, Austria
